# Analysis of Host Gene Expression Profile in HIV-1 and HIV-2 Infected T-Cells

**DOI:** 10.1371/journal.pone.0147421

**Published:** 2016-01-28

**Authors:** Krishnakumar Devadas, Santanu Biswas, Mohan Haleyurgirisetty, Owen Wood, Viswanath Ragupathy, Sherwin Lee, Indira Hewlett

**Affiliations:** Laboratory of Molecular Virology, Division of Emerging and Transfusion Transmitted Diseases, Center for Biologics Evaluation and Research, Food and Drug Administration, 10903 New Hampshire Ave, Silver Spring, Maryland, 20993–0002, United States of America; George Mason University, UNITED STATES

## Abstract

HIV replication is closely regulated by a complex pathway of host factors, many of them being determinants of cell tropism and host susceptibility to HIV infection. These host factors are known to exert a positive or negative influence on the replication of the two major types of HIV, HIV-1 and HIV-2, thereby modulating virus infectivity, host response to infection and ultimately disease progression profiles characteristic of these two types. Understanding the differential regulation of host cellular factors in response to HIV-1 and HIV-2 infections will help us to understand the apparent differences in rates of disease progression and pathogenesis. This knowledge would aid in the discovery of new biomarkers that may serve as novel targets for therapy and diagnosis. The objective of this study was to determine the differential expression of host genes in response to HIV-1/HIV-2 infection. To achieve this, we analyzed the effects of HIV-1 (MN) and HIV-2 (ROD) infection on the expression of host factors in PBMC at the RNA level using the Agilent Whole Human Genome Oligo Microarray. Differentially expressed genes were identified and their biological functions determined. Host gene expression profiles were significantly changed. Gene expression profiling analysis identified a subset of differentially expressed genes in HIV-1 and HIV-2 infected cells. Genes involved in cellular metabolism, apoptosis, immune cell proliferation and activation, cytokines, chemokines, and transcription factors were differentially expressed in HIV-1 infected cells. Relatively few genes were differentially expressed in cells infected with HIV-2.

## Introduction

A multitude of factors including viral diversity, host genetics and immunological factors contribute to pathogenesis and disease progression in HIV infected individuals. HIV infection is also known to impact host gene expression and cause profound changes to cellular physiology and metabolism [1–4]. However, the impact of host factors on pathogenesis and disease progression in infected individuals is not very well understood. HIV replication is closely regulated by a complex pathway of host factors and many of these host factors are determinants of cell tropism and host range of HIV and could positively or negatively regulate HIV replication. Indeed, several reports have shown that interaction of virus-encoded proteins with cellular proteins play a crucial role in viral replication and host determination. To date, over 250 host factors have been identified that modulate viral expression and disease progression [5–7]. Although many host factors have been identified, the regulatory mechanism of host factors on HIV life cycle is still not fully understood. Understanding the mechanisms that contribute to different outcomes of viral infection are important aspects in studying HIV pathogenesis and may contribute to the identification of new biomarkers of infection that could serve as new targets for therapy and aid in diagnosis. Therefore, it is important to determine the impact of host regulatory factors and cellular determinants on the replication kinetics of emerging, diverse HIV variants.

HIV is characterized by a high degree of genetic variation and includes two major types HIV-1 and HIV-2, and at least 11 different subtypes (A-K) of HIV-1, representing the major group M, and viruses representing the minor groups 0, N and P are responsible for the AIDS pandemic. HIV-1 and HIV-2 are closely related retroviruses that share many similar traits like modes of transmission, viral replication and pathogenesis. However, major differences exist between the clinical outcomes presented by the two viruses. Clinically, HIV-2 patients have a higher CD4 cell count at the time of AIDS, and generally have a longer survival after AIDS. Most people infected with HIV-2 do not progress to disease, even though the minority who do progress cannot be distinguished clinically from HIV-1-infected patients. HIV-1 is more pathogenic than HIV-2, with higher measurable levels of plasma viremia, however, the exact mechanisms contributing to these differences is not completely understood. Several studies have reported that pathogenesis of HIV-1 and HIV-2 differs at the cellular level with respect to virus infectivity, viral replication and host response to infection [3, 4, 8, 9]. Our published studies have shown that significant variations in cytopathic effects occur following *in vitro* infection with primary isolates of HIV-1 or HIV-2 subtypes in PBMC [9]. Preliminary findings using PBMC or Jurkat cells infected with HIV-1 or HIV-2 indicated that HIV-1 infection led to significant decreased cell growth and that there were differences between HIV-1 and HIV-2 infection at the molecular level that involved NF-kappa B and MAPKs signaling pathways and specific apoptosis-related proteins [10–13]. Thus, we undertook this study to understand the variations of cellular responses between the two HIV infections and to delineate the mechanisms of their pathogenesis. These studies should enable identification of new diagnostic and predictive biomarkers that could be putative targets for therapy.

With the advent of new high-throughput techniques, like next generation sequencing and microarray analysis, it is possible to obtain and analyze information on gene expression patterns comparing different groups of samples. Using these methods, there are many reports of *in vivo* or *in vitro* studies identifying differential gene expression profiles from HIV-1 infected patient samples and different cell types [14–21]. However, there is a lack of information about the profile changes induced by different HIV strains. Therefore, we initiated several studies to determine the differential expression of host genes in response to HIV-1/HIV-2 infection in order to understand mechanisms underlying delayed disease progression seen in most people infected with HIV-2. To achieve this, we analyzed the effects of HIV-1 (MN) and HIV-2 (ROD) infection on the expression of host factors in PBMC at the RNA level using the Agilent Whole Human Genome Oligo Microarray. Differentially expressed genes were identified and their biological functions determined. Host gene expression profiles were significantly changed at the time point tested. The gene expression profiling analysis identified a subset of differentially expressed genes in HIV-1 and HIV-2 infected cells. Genes involved in cellular metabolism, cytokines and chemokines, apoptosis, B and T cell activation and proliferation, cellular receptors and transcription factors were differentially expressed in HIV-1 infected cells. Relatively few genes were differentially expressed in cells infected with HIV-2. Notably, genes for proteins like heat shock protein (HSPA6), Splicing factor, arginine/serine-rich 9 (SFRS9), and keratin (KRT1) were differentially expressed in HIV-2 infected cells.

## Materials and Methods

### Peripheral blood mononuclear blood cells isolation and culture

Buffy coats from individual seronegative donors were provided by the NIH Blood bank. A categorical exemption is in place for CBER / FDA for experimental studies by CBER/FDA researchers using existing, deidentified samples of blood and /or blood products originally obtained under the NIH IRB-approved protocol and consent form 99-CC-0168. Written informed consent was obtained from healthy, normal blood donors for publication of this Case Report and any accompanying images according to the ethical principles of international ethical guidelines for biomedical research involving human subjects. This study was approved by the NIH ethics committee (study number: 99-CC-0168, PI: Susan F. Leitman, M.D.). Peripheral blood mononuclear cells (PBMC) were isolated from these buffy coats by a single step Ficoll-Hypaque density gradient method. Freshly isolated PBMC were stimulated for 72 h by culturing in RPMI-1640 medium supplemented with 10% heat inactivated FBS, penicillin-streptomycin, 10 U/ml IL-2 and 10μg/ml of PHA. The PBMC were cultured for 24 h post stimulation in regular media prior to infection. Jurkat cell line [22] was cultured in RPMI-1640 medium supplemented with 10% heat inactivated FBS, 1% penicillin, and 1% streptomycin.

### Viruses and infection

Jurkat cells were seeded at 1×10^6^ cells/ml for 24 h, and infected with known amounts (10ng/ml p24 units per 10^6^ cells) of HIV-1 (MN) and HIV-2 (ROD) and cultured for different days as indicated. PBMC cells were infected with known amounts (5ng/ml p24 units per 10^6^ cells) of HIV-1 (MN) and HIV-2 (ROD). Two hours post-infection, the tubes were washed with PBS to remove un-adsorbed virus and cells were fed with complete RPMI (RPMI 1640, 10% FBS, 1% penicillin–streptomycin supplemented with IL-2) and cultured in T75 flask. The cells were harvested at different times points as indicated (day 7 and day 15).

### RNA extraction

Total RNA was extracted from infected and uninfected cells using miRNeasy total RNA isolation kit (QIAgen) according to the manufacturer’s protocol. Briefly cells were lysed with 700μl of QIAzol lysis reagent. DNA was sheared using QIAshredder columns (QIAgen) and total RNA was extracted from this lysate using the RNeasy^®^ Mini column (QIAgen) according to the manufacturer’s protocol. RNA amount was estimated by spectrophotometry using the Nanodrop 1000 (Thermo Fisher).

### Agilent whole genome oligo microarray

Agilent microarray hybridization and analysis was carried out by ArrayStar Inc., MD. Briefly, the RNA samples that passed quality control on the Nanodrop ND-1000 and Bioanalyser 2100 were amplified and labeled using the Agilent Quick Amp Labeling Kit and hybridized to Agilent whole genome oligo microarray in Agilent’s SureHyb hybridization chambers. After hybridization and washing, the processed slides were scanned using the Agilent DNA microarray scanner following Agilent Technologies’ guidelines. The resulting.txt files extracted from Agilent Feature Extraction Software (version 10.5.1.1) were imported into the Agilent GeneSpring GX software (version11.0) for further analysis. The microarray data sets were normalized in GeneSpring GX using the Agilent FE one-color scenario. After quantile normalization of the raw data, genes that were categorized as Detected (“All Targets Value”) and had flags in at least 20 out of 24 samples were chosen for further data analysis. Differentially expressed genes were identified through Fold-change screening. The p-value was calculated using t-test. The p-values were corrected for multiple comparisons using the Benjamini & Hochberg false discovery rate method [23]. The analysis of Gene Ontology (GO) and Pathway is based on Fisher’s exact test.

### Gene co-expression network analysis

Gene co-expression networks were constructed for each of the top 25 upregulated and 25 down regulated differentially expressed genes in HIV-1 infected PBMC or in HIV-2 infected PBMC, by screening for significant gene to gene interactions using the online Gene MANIA program (http://www.genemania.org/) [24, 25]. The Gene MANIA program is an extensive database that uses a very large set of previously discovered functional association data and networks to find other genes that are related to a set of input genes. The GO term (biological process)-based weighting was used to determine the correlation coefficient between a differentially expressed input gene and related genes in its network to find the top 100 related genes with at most 20 attributes connecting the genes. Distinct functional modules of the top differentially expressed genes and additional related co-expressed were constructed from the top 25 upregulated and 25 down regulated genes with the top 100 connected edges in the co-expression networks using the Cytoscape vs.3.2.1 software (http://www.cytoscape.org/) plugin ClusterMaker using the community clustering application (GLay) [26, 27].

### cDNA synthesis and quantitative real-time PCR

To validate the microarray results derived from ArrayStar Inc., we tested the expression of selected genes by RT-PCR using RNA samples isolated from 5 independent donors. Real-time -PCR was performed using the two-step RT-PCR with SYBR-Green and ROX (Invitrogen) according to manufacturer's protocols. RNA from uninfected and infected PBMC was reverse transcribed with Superscript^®^ III First-Strand Synthesis SuperMix (Invitrogen). The resulting cDNA was then amplified by gene specific primers for real-time PCR by the SYBR-Green PCR mix (Invitrogen) which consisted of AmpliTaq^®^ Fast DNA Polymerase, UP (Ultra Pure), SYBR^®^ Green I dye, ROX^TM^ dye passive reference, Tris-HCl, KCl, 6 mM MgCl2, 400 μM dGTP, 400 μM dATP, 400 μM dCTP, 800 μM dUTP, uracil DNA glycosylase (UDG), and 20μM forward and reverse primers. The program for thermal cycling was 20 s at 95°C followed by 40 cycles of 3 s at 95°C, 30 s at 57°C. Housekeeping gene, GAPDH, was used as an endogenous control for each donor. Each sample was run in triplicate to ensure accurate fold change estimation. Relative gene expression was calculated by the ΔΔCt method. Briefly, Ct values for the gene of interest and GAPDH was obtained from each donor RNA and the Ct value of the gene-of interest was normalized to the Ct value of GAPDH. Final results are expressed as n-fold difference in expression of gene-of-interest relative to GAPDH gene for infected and uninfected as n-fold = 2^ − (ΔCt infected −ΔCt uninfected).

### Western Blot

Cultures of PBMC were harvested and washed in ice-cold PBS three times. Proteins were isolated from the cells with RIPA buffer (Thermo Scientific cat # 89901). Total protein was quantitated using Pierce® BCA protein assay kit- reducing agent compatible (Thermo scientific). Equal amounts of protein (5μg) were heated (70°C for 10 minutes) in the NuPAGE® LDS sample buffer and NuPAGE®reducing agent and separated on NuPAGE® Bis-Tris Gel (4–12%) (Life technologies) and blotted onto Amersham Hybond ECL Nitrocellulose membrane. Cellular proteins were detected with the primary mouse monoclonal antibody raised against recombinant 3PGDH of human origin (Santa Cruz Biotechnology, Inc., Cat # sc-100317), rabbit polyclonal antibody raised against synthetic PSAT1 peptide of human origin (Santa Cruz Biotechnology, Inc., Cat # sc-133929) and β-Actin rabbit monoclonal antibody (Cell Signaling). Proteins were visualized using an ECL western blotting analysis system (GE Healthcare).

## Results

### Microarray and differential gene expression analysis results

**Fig 1:**
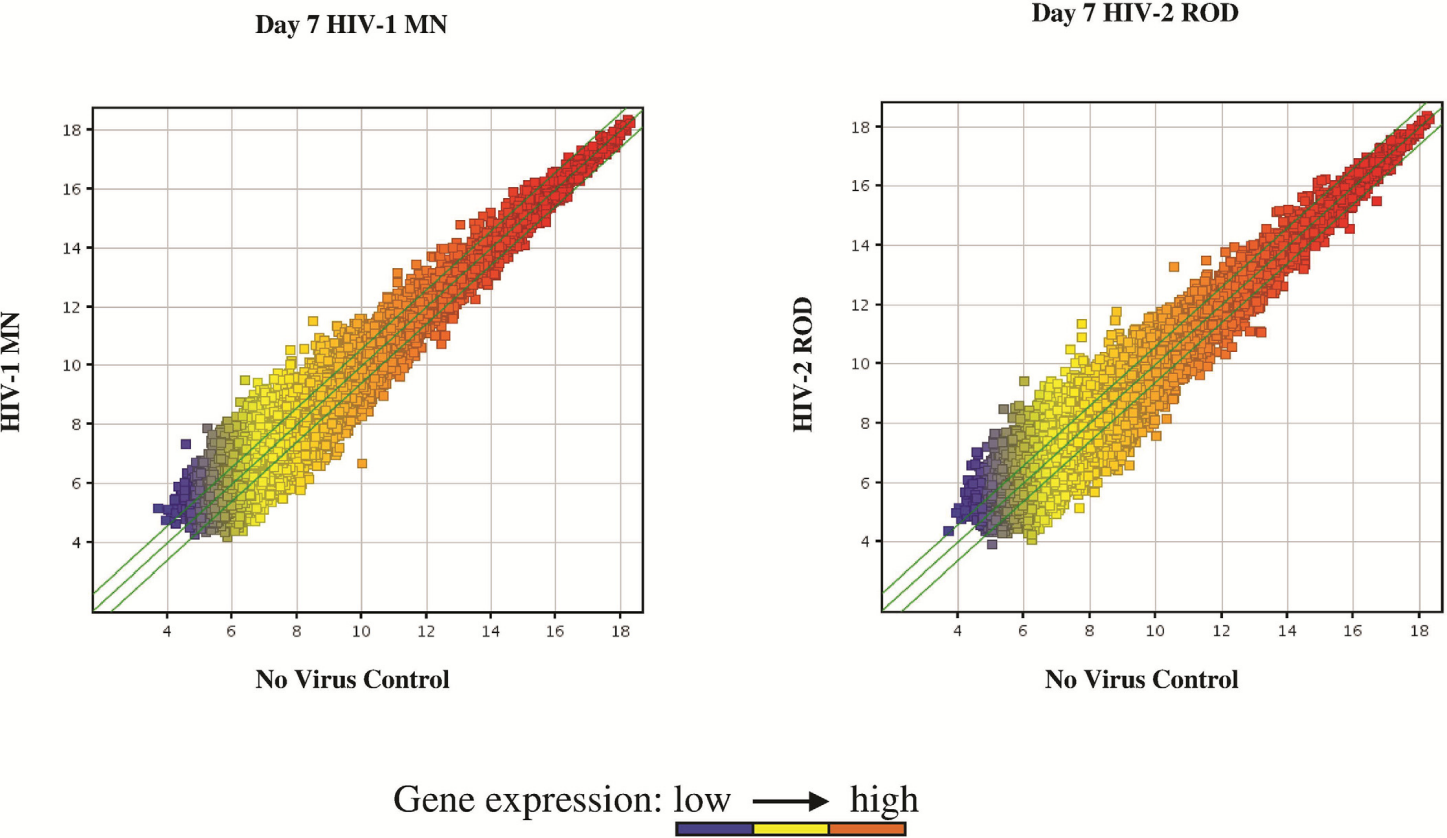
Scatter plot of genes expressed in HIV-1 or HIV-2 infected cells, Day 7 post infection. The values of X and Y axes in the Scatter-Plot are the normalized signal values of the averaged normalized signal values of the compared groups. The green lines are Fold Change Lines (The default fold change value is 1.5). The genes above the top green line and below the bottom green line indicated more than 1.5 fold change of genes between two samples or groups.

**Fig 2:**
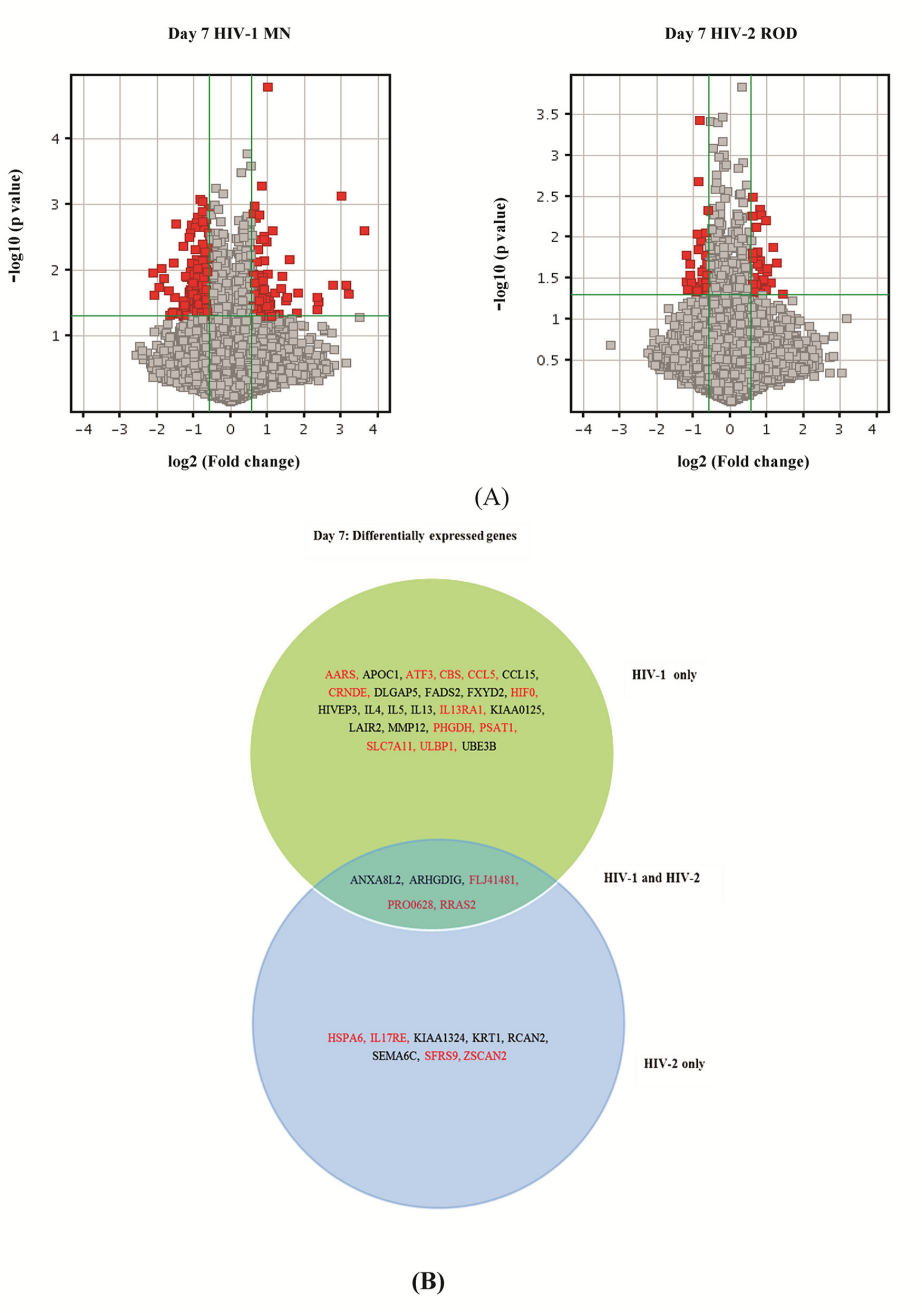
Differentially expressed genes in HIV-1 or HIV-2 infected cells. (A) Volcano Plot of differentially expressed genes in HIV-1 or HIV-2 infected cells, Day 7 post infection. The vertical lines correspond to 1.5-fold up and down and the horizontal line represents a P-value of 0.05. (B) Venn diagram of a subset of differentially expressed genes in HIV-1 or HIV-2 infected cells, Day 7 post infection. The genes depicted in red represent up regulated genes and genes depicted in black represent down regulated genes.

RNA samples from PBMC isolated from 3 independent donors productively infected with HIV-1 MN or HIV-2 ROD (Table 1) were used to obtain gene expression data from Agilent microarrays. The Agilent microarray used contained 43,377 probe sets corresponding to approximately 30,000 genes described for whole human genome. Agilent Feature Extraction software (version 10.7.3.1) was used to analyze acquired array images. Quantile normalization and subsequent data processing were performed using the GeneSpring GX v11.5.1 software (Agilent Technologies). The Scatter-Plot is a visualization method used for assessing the gene expression variation (or reproducibility) between arrays. The values of X and Y axes in the Scatter-Plot are the normalized signal values of the averaged normalized signal values of the compared groups (Fig 1). The green lines are Fold Change Lines (The default fold change value is 1.5). The genes above the top green line and below the bottom green line indicated more than 1.5 fold change of genes between two groups. Differentially expressed genes were identified through Fold Change filtering and Volcano Plot filtering (Fig 2A). To identify differentially expressed genes a Fold Change filtering between two samples was performed. The default threshold was set at Fold Change ≥1.5. To identify differentially expressed genes with statistical significance, a Volcano Plot filtering between two groups was performed using a default threshold of Fold Change ≥1.5, P-value ≤0.05. The p-values were adjusted for multiple comparisons by using the Benjamini & Hochberg false discovery rate method [23]. Analysis revealed a number of differentially expressed genes between HIV-1 MN and HIV-2 ROD infected cells at the time point tested (see S1 File). A total of 116 genes were found to be differentially up-regulated and 200 genes were down-regulated in HIV-1 infected samples compared to the uninfected control at day 7. Whereas, at day 7 post-infection, a substantially lower number of genes were found to be differentially up-regulated (21 genes) or down-regulated (36 genes) in HIV-2 infected cells, indicating a different pattern of gene expression consistent with a lower level of cell activity compared to HIV-1 infected cells. A few of the differentially expressed genes were common to both HIV-1 and HIV-2 infected PBMC at day 7 post infection (Fig 2B). Additional analysis comparing the differentially expressed genes from our data set to data sets from clinical and experimental HIV studies deposited in the Gene Expression Omnibus data base were carried out. Comparison of our data with the data from clinical HIV studies identified a few common genes that were differentially expressed (S1 Table). Comparison of our data set to the differentially expressed genes between PBMCs isolated from 22 seropositive persons and 12 seronegative persons shown in GSE2171 data set [18] revealed that genes like ATF3, HIF0, NAMPT, and RGS16 were commonly up-regulated and genes like ZBTB32, CDC25C, ME3, FABP5, APOC1 and RAD54L were down regulated. Similarly, comparison of our data set to the differentially expressed genes from CD4+ and CD8+ T cells from HIV patients identified in GSE6740 data set [28] demonstrated that common genes like ANKH, IL13RA1, NAMPT, B3GALT2, CREBBP, and TGIF1 were upregulated and other genes like ZBTB32, MRPS14, RBM8A, FABP5, HIVEP3, SLC29A1 and DLGAP5 were commonly down regulated.

**Table 1:** Quantification of HIV-1 (P-24) and HIV-2 (P-26) antigens.

Donor	HIV-1 p24 (pg/ml)	HIV-2 p26 (pg/ml)
No. 1	1909	17683.965
No. 2	5649	46071.77
No. 3	1803	41414.58

HIV-1 replication was quantitated by measuring HIV-1 p24 using the Alliance HIV-1 p24 Antigen ELISA Kit (Perkin Elmer) and HIV-2 replication was quantitated by measuring HIV-2 p26 using the RETRO-TEK SIV p27 Antigen ELISA Kit (ZeptoMetrix).

### Functional analysis: GO and Pathway enrichment analysis results of differentially expressed genes

**Fig 3:**
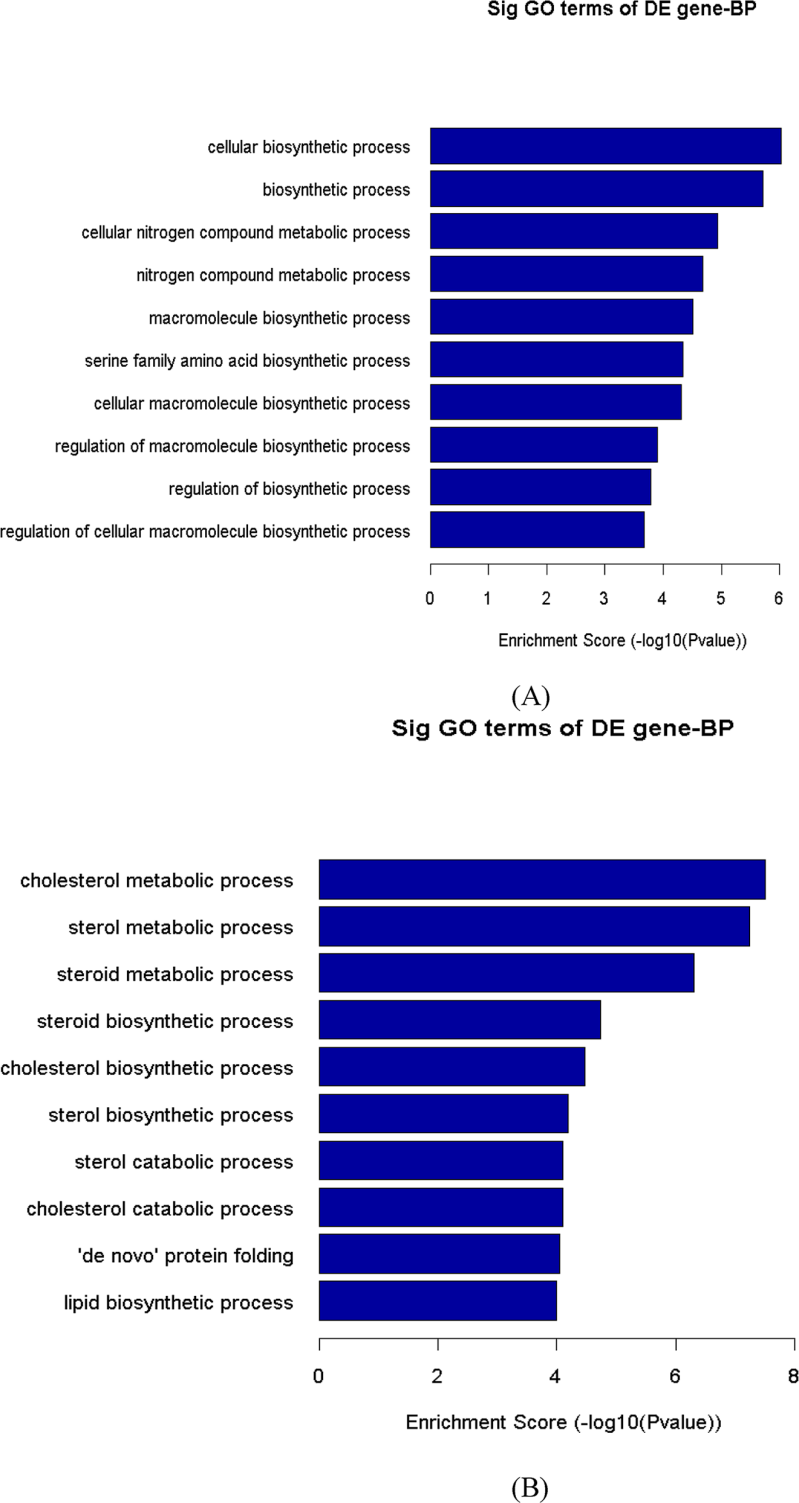
Gene Ontology (GO) analysis of differentially expressed genes in HIV-1 MN infected cells. The chart shows the top ten counts of the significant enrichment terms. The count describes the number of differentially expressed genes associated with the listed GOID. The top GO (biological process) terms that occur in the differently expressed genes from PBMCs infected cells with HIV-1 MN are shown. The bar plot shows Fold Enrichment value of the significant enrichment terms. Pathway analysis for differentially expressed (A) up regulated genes and (B) down regulated genes.

**Fig 4:**
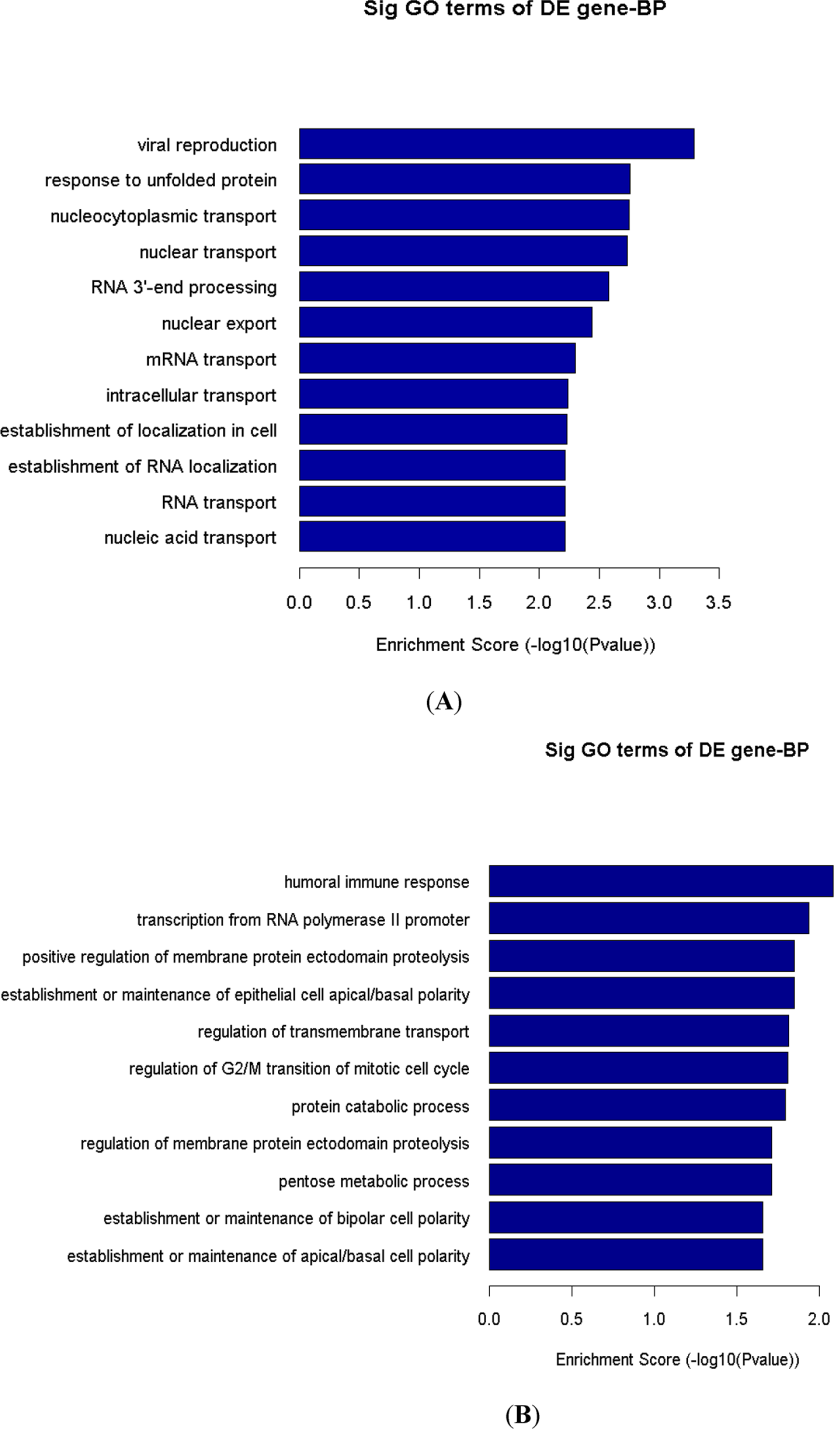
Gene Ontology (GO) analysis of differentially expressed genes in HIV-2 ROD infected cells. The top GO (biological process) terms that occur in the differently expressed genes in PBMCs infected cells with HIV-2 ROD are shown. The bar plot shows Fold Enrichment value of the significant enrichment terms. Pathway analysis for differentially expressed (A) up regulated genes and (B) down regulated genes.

The differentially expressed genes were subjected to functional analysis using the Gene Ontology database (GO). GO analysis helps to associate the each of differentially expressed gene with its function based on GO categories and facilitates the understanding of the intricate network of gene interactions and its impact on viral replication. The GO categories are derived from Gene Ontology database (http://www.geneontology.org) which comprises of three structured networks of defined terms that describe gene product attributes like different biological processes, cellular components and molecular functions describing involved metabolic pathways. Fisher’s exact test was used to determine if there was more overlap between the differentially expressed genes and the GO annotation list than would be expected by chance. The significance of GO Term enrichment in the differentially expressed gene list was based on a P-value ≤ 0.05. From this analysis relevant data (see S2 File) for the differentially expressed genes about the subcellular distribution (cellular component), the association with different biological processes, and the impact on metabolic pathways and molecular functions could be deduced. Analysis of the data revealed considerable variation in the sub-cellular distribution of the differentially expressed genes between HIV-1 and HIV-2 infected cells at the time point (day 7) tested. Differentially expressed genes in HIV-1 or HIV-2 infected cells at day 7 post-infection exhibited a similar localization pattern. The genes identified on day 7 post-infection in HIV-1/HIV-2 infected cells were predominantly sequestered within the cytoplasm and associated with organelle membranes like the endoplasmic reticulum and sarcoplasmic reticulum, plasma membrane and lipid-protein complexes. In addition, several genes were localized to the extracellular space. GO analysis revealed significant differences between genes differentially expressed on day 7 post-infection in HIV-1 and HIV-2 infected cells for biological processes. The biological processes for genes differentially expressed in HIV-1 infected cells include genes involved in apoptosis, regulation of transcription, cell cycle, biosynthesis, B-cell /T-cell activation and proliferation, lipid and steroid metabolism were found to be significantly down-regulated (Fig 3). Whereas, genes involved in cellular biosynthesis and amino acid metabolism, gene regulation and transcription were found to be up-regulated (Fig 3). The biological processes implicated for the differentially expressed genes in HIV-2 infected cells were predominantly genes involved in biological regulation, humoral immune response, viral reproduction and nuclear transport, signal transduction and organic acid metabolism (Fig 4). Analysis of the implicated molecular functions of the differentially expressed genes in HIV-1 infected cells on day 7 post-infection revealed that the gene functions were related to protein and nucleic acid binding, transcription factor binding, ATPase and GTPase activity, cellular transport and apoptosis activator activity. Comparatively a lower number of genes were differentially expressed in HIV-2 infected cells at day 7 post-infection. The molecular functions of the differentially expressed genes were carbohydrate kinase activity, oxidoreductase activity, catalytic activity, kinase activity, protein binding activity and transcription repressor activity. Specific metabolic pathways were analyzed using the KEGG database. In HIV-1 infected cells the differentially expressed genes on day 7 post-infection were mainly related to steroid biosynthetic pathway, terpenoid backbone synthesis, biosynthesis of unsaturated fatty acids, glycine, serine and threonine metabolism pathways (Table 2). In contrast, in HIV-2 infected cells the differentially expressed genes were associated predominantly with pathways involved in Neuroactive ligand-receptor interaction, arachidonic acid metabolism pathway, fat digestion and absorption, antigen processing and presentation pathway and Hematopoietic cell lineage pathway (Table 2).

**Table 2:** KEGG Pathway analysis of the differentially expressed genes.

Virus	Regulation	Pathway ID	Definition	Fisher p value	Selection Counts	Selection Size	Count	Size	FDR	Enrichment Score	Genes
HIV-1	down	hsa00900	Terpenoid backbone biosynthesis	0.00056	4	165	15	6080	0.015283	3.247976	ACAT2,FDPS,MVK,PDSS1
HIV-1	down	hsa00100	Steroid biosynthesis	0.00147	4	165	19	6080	0.03316	2.831573	CYP51A1,DHCR24,DHCR7,EBP
HIV-1	down	hsa01040	Biosynthesis of unsaturated fatty acids	0.00218	4	165	21	6080	0.040121	2.661269	ACOT7,FADS1,FADS2,SCD
HIV-1	down	hsa04975	Fat digestion and absorption	0.01229	13	890	46	6080	0.199625	1.910125	ABCG5,AGPAT2,APOA1,CEL,MOGAT3,NPC1L1,PLA2G1B,PLA2G2A,PLA2G4E,PLA2G5,PLA2G6,PNLIPRP1,SCARB1
HIV-1	down	hsa05310	Asthma	0.00929	4	165	31	6080	0.089598	2.031907	HLA-DOA,IL10,IL4,IL5
HIV-1	up	hsa00260	Glycine, serine and threonine metabolism—Homo sapiens	0.00237	5	182	32	6080	0.149666	2.624423	CBS,PHGDH,PSAT1,PSPH,SHMT2
HIV-1	up	hsa05166	HTLV-I infection	0.03023	12	149	267	6080	0.329466	1.519535	AKT2,ATF3,CANX,DLG1,E2F3,FOS,ICAM1,IL15RA,IL6,MAP3K3,MSX2,RRAS2
HIV-2	up	hsa04141	Protein processing in endoplasmic reticulum	0.00624	6	61	167	6080	0.09217	2.204164	CANX,HSP90AA1,HSPA1B,HSPA6,PDIA4,STT3A
HIV-2	up	hsa03040	Spliceosome	0.00582	10	187	128	6080	0.063596	2.234897	ACIN1,HNRNPA1,HNRNPA1L2,PRPF38B,RBM25,RBMXL1,SF3B1,SNRNP40,SNRPE,THOC1

Pathway ID: Pathway identifiers used in KEGG. Definition: The definition of the Pathway ID. Fisher-P value: The enrichment p-value of the Pathway ID using Fisher’s exact test. Selection Counts: Count of the differentially expressed gene entities directly associated with the listed Pathway ID. Selection Size: Total number of the differentially expressed gene entities. Count: Count of the chosen background population gene entities associated with the listed Pathway ID. Size: Total number of chosen background population gene entities. FDR: The false discover rate of the Pathway ID. Enrichment Score: The Enrichment Score value of the Pathway ID, it equals "-log10 (P value)”. GENES: The genes which are from the dataset associated with the Pathway ID.

### Gene co-expression network analysis of differently expressed genes

**Fig 5:**
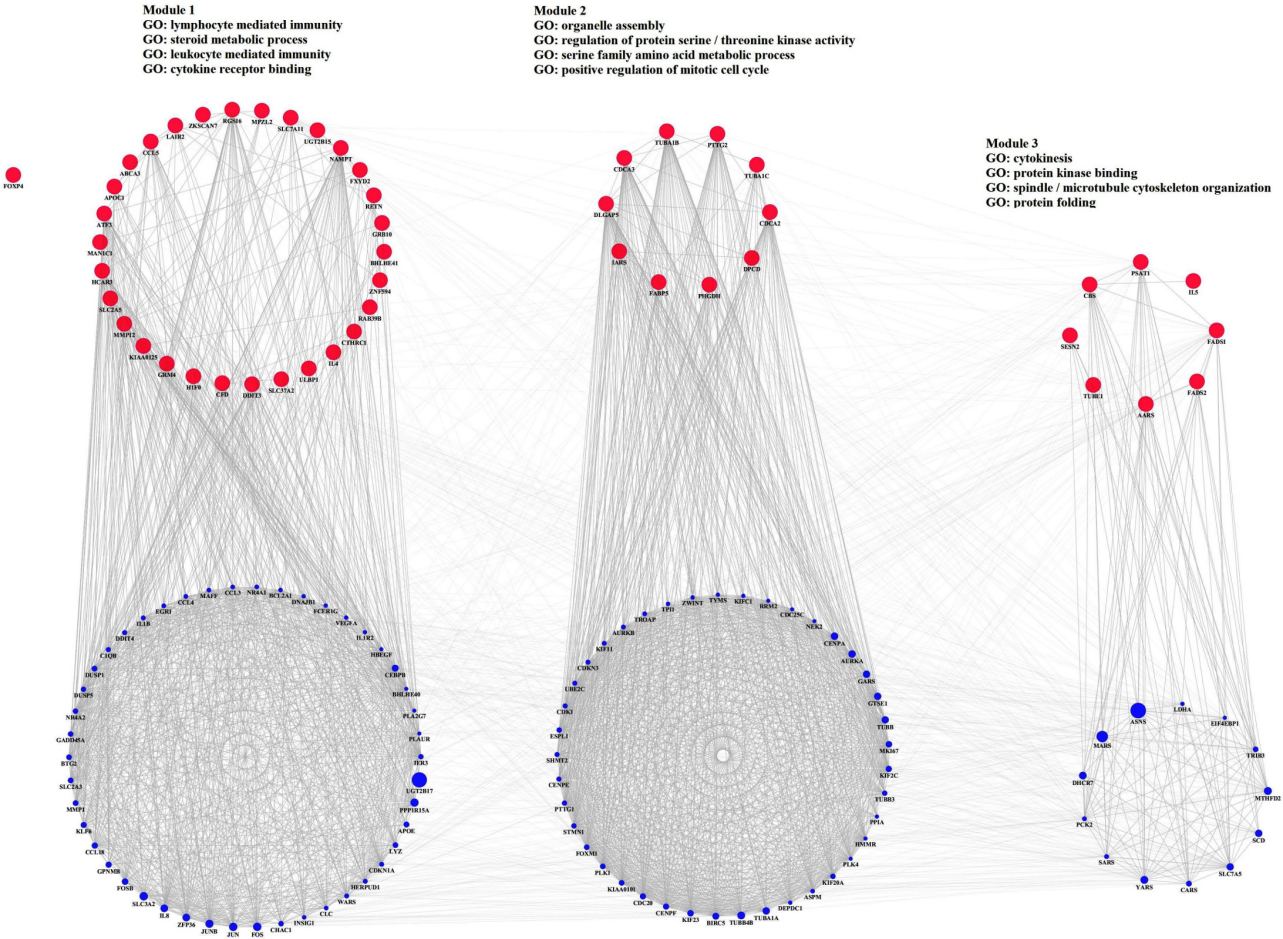
Identification of functional modules in gene co-expression network. From the co-expression network constructed using the top 25 up-regulated and 25 down-regulated differentially expressed genes identified in PBMC infected with HIV-1 as input genes, three functional modules were clustered. The color of the node signifies the following: Pink circle denotes input genes, Blue circle denotes co-expressed genes.

**Fig 6:**
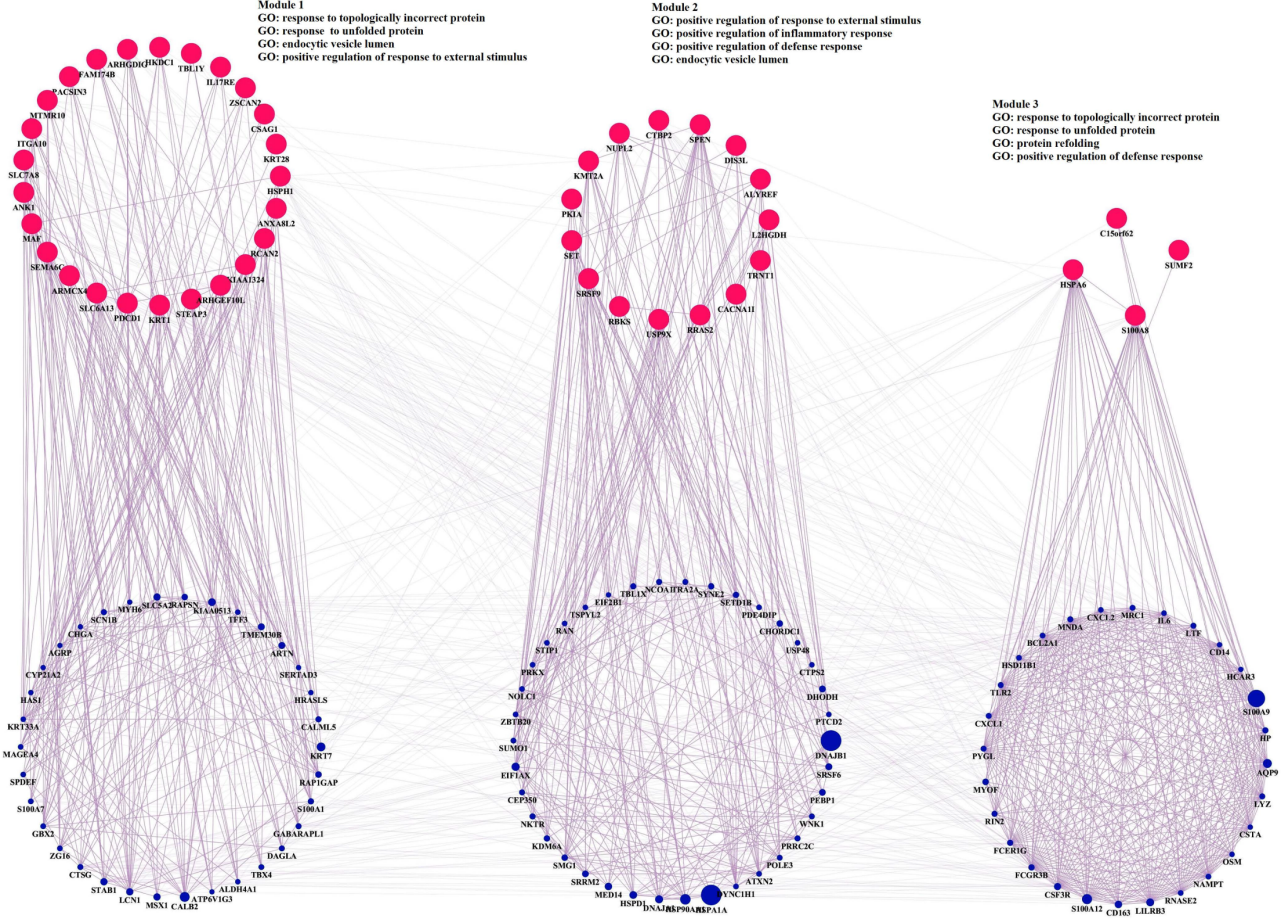
From the co-expression network constructed using the top 25 up-regulated and 25 down-regulated differentially expressed genes identified in PBMC infected with HIV-2 as input genes, three functional modules were clustered. The color of the node signifies the following: Pink circle denotes input genes, Blue circle denotes co-expressed genes.

To interpret the biological meaning of the differentially expressed genes, a co-expression network was constructed using the top 25 up-regulated and 25 down-regulated differentially expressed genes identified in PBMC infected with HIV-1 or HIV-2 as input genes. The co-expression network analyses of the differentially expressed genes from HIV-1 infected PBMC identified significant interactions composed of 149 nodes and 2602 edges. Genes from the co-expression network of the differentially expressed genes in HIV-1 infected PBMC such as asparagine synthetase (ASNS), UDP glucuronosyltransferase 2 family, polypeptide B17 (UGT2B17), methionyl-tRNA synthetase (MARS), solute carrier family 3 (amino acid transporter heavy chain), member 2 (SLC3A2), FBJ murine osteosarcoma viral oncogene homolog (FOS), jun proto-oncogene (JUN), protein phosphatase 1, regulatory subunit 15A (PPP1R15A), jun B proto-oncogene (JUNB), methylenetetrahydrofolate dehydrogenase (NADP+ dependent) 2, methenyltetrahydrofolate cyclohydrolase (MTHFD2), and tyrosyl-tRNA synthetase (YARS) were identified as genes with the most connected edges. The distinct functional modules of the differently expressed genes and their interacting genes were identified by implementation of the Girvan-Newman fast greedy algorithm of the GLay Cytoscape plugin (Fig 5). Three functional clusters / modules were identified. Among the modules, “Module 1”, which has the largest size, was significantly enriched by the GO biological process terms such as lymphocyte mediated immunity, steroid metabolic process, leukocyte mediated immunity and cytokine receptor binding. Similarly, the gene co-expression networks constructed for the differentially expressed genes in HIV-2 infected PBMC identified significant interactions composed of 144 nodes and 1247 edges. Genes with the most connected edges were identified as DnaJ (Hsp40) homolog, subfamily B, member 1 (DNAJB1), heat shock 70kDa protein 1A (HSPA1A), S100 calcium binding protein A9 (S100A9), heat shock protein 90kDa alpha (cytosolic), class A member 1 (HSP90AA1), S100 calcium binding protein A12 (S100A12), calbindin 2 (CALB2), aquaporin 9 (AQP9), keratin 7 (KRT7), eukaryotic translation initiation factor 1A, X-linked (EIF1AX), and DnaJ (Hsp40) homolog, subfamily A, member 1 (DNAJA1) from the co-expression networks constructed with the differentially expressed genes from HIV-2 infected PBMC. Clustering analysis identified 3 functional modules (Fig 6). Module 1 comprised of the maximum number of genes was significantly enriched by the GO biological process terms such as response to topologically incorrect protein, response to unfolded protein, endocytic vesicle lumen, and positive regulation of response to external stimulus.

### Validation of microarray data by real-time PCR

**Fig 7:**
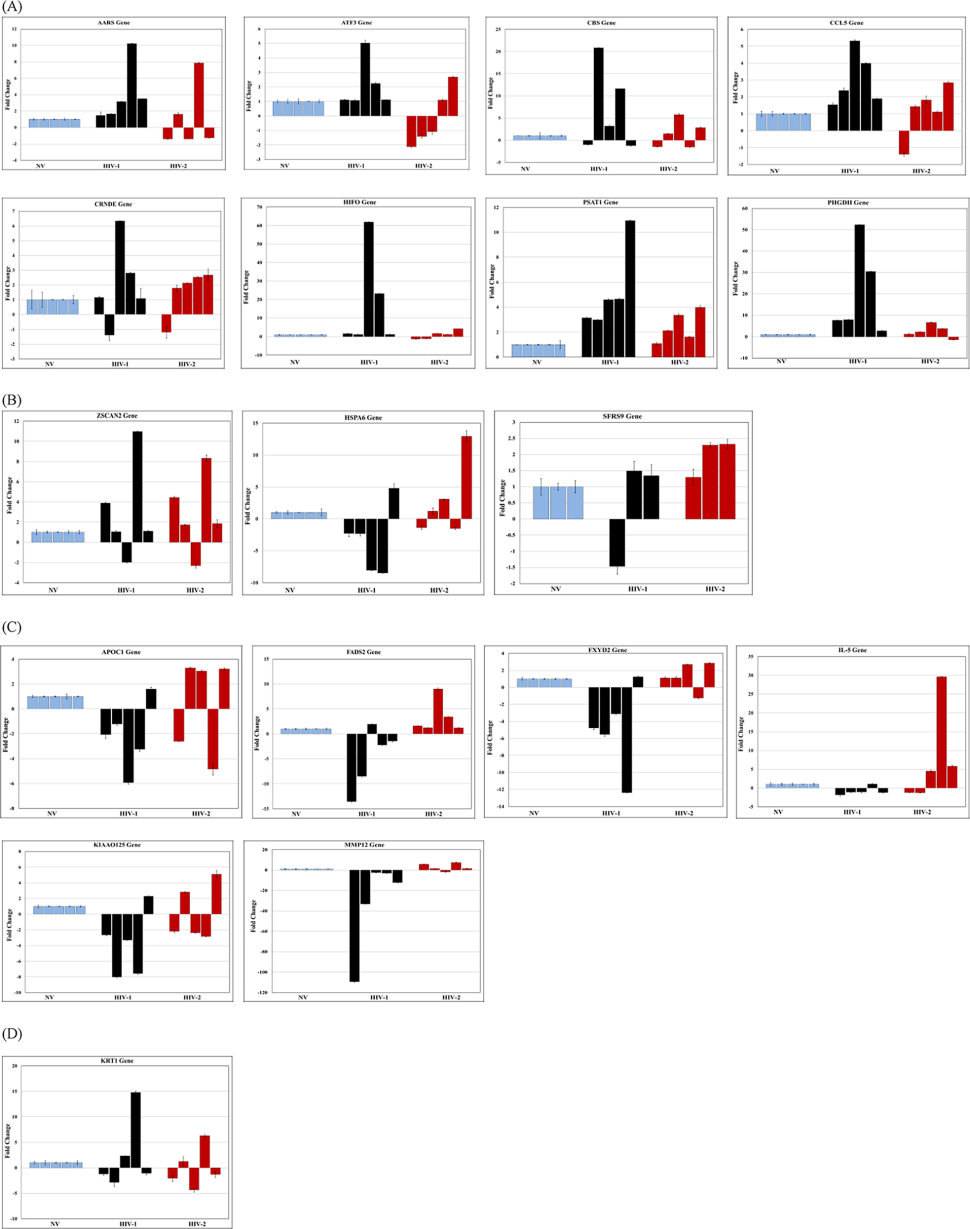
Real-time PCR detection of host genes in PBMCs infected with HIV1 or HIV-2 seven days post infection. The RT-PCR results from RNA isolated from PBMCs from five different donors infected with HIV-1(MN) and HIV-2 (ROD) to validate microarray data *(*A) genes differentially up regulated only in HIV-1 infected cells, (B) genes differentially up regulated only in HIV-2 infected cells, (C) genes differentially down regulated only in HIV-1 infected cells and (D) genes differentially down regulated only in HIV-2 infected cells. The data depicts results from 3 independent experiments. Each experimental sample subjected to real-time PCR amplification was normalized relative to the endogenous GAPDH control and the relative amount of target gene quantitated. Final results are expressed as n-fold difference in expression of gene-of-interest relative to GAPDH gene for infected and uninfected cells as n-fold = 2^ − (ΔCt infected −ΔCt uninfected). Each sample was run in triplicate to ensure accurate fold change estimation and the results expressed as mean ± SEM.

**Fig 8:**
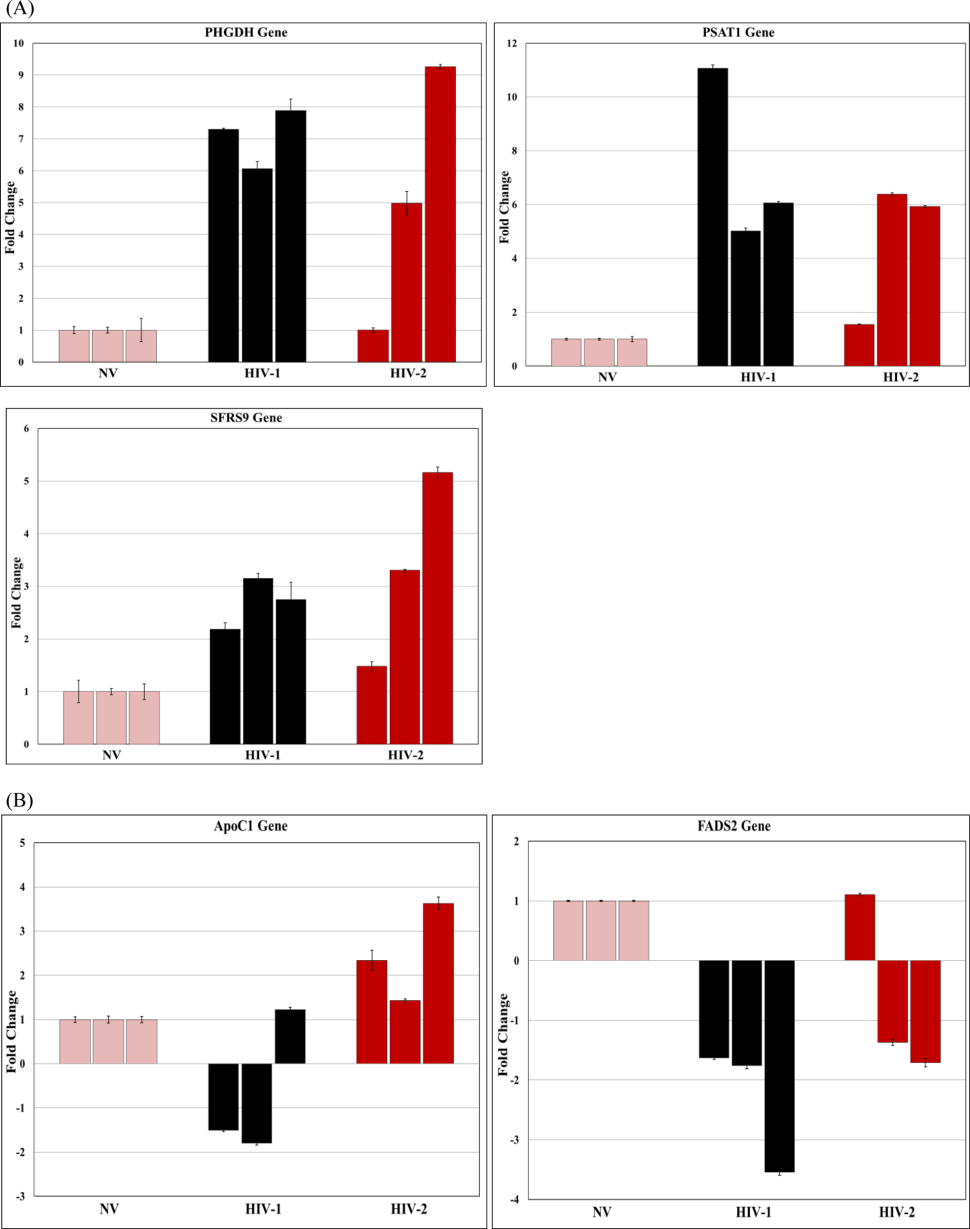
Real-time PCR detection of host genes in Jurkat cells infected with HIV-1 or HIV-2 seven days post infection. The RT-PCR results from RNA isolated from Jurkat cells infected with HIV-1(MN) and HIV-2 (ROD) to validate microarray data *(*A) genes differentially up regulated in HIV-1 and HIV-2 infected cells and (B) genes differentially down regulated in HIV-1 and HIV-2 infected cells. The data depicts results from 3 independent experiments. Each experimental sample subjected to real-time PCR amplification was normalized relative to the endogenous GAPDH control and the relative amount of target gene quantitated. Final results are expressed as n-fold difference in expression of gene-of-interest relative to GAPDH gene for infected and uninfected cells as n-fold = 2^ − (ΔCt infected −ΔCt uninfected). Each sample was run in triplicate to ensure accurate fold change estimation and the results expressed as mean ± SEM.

**Fig 9:**
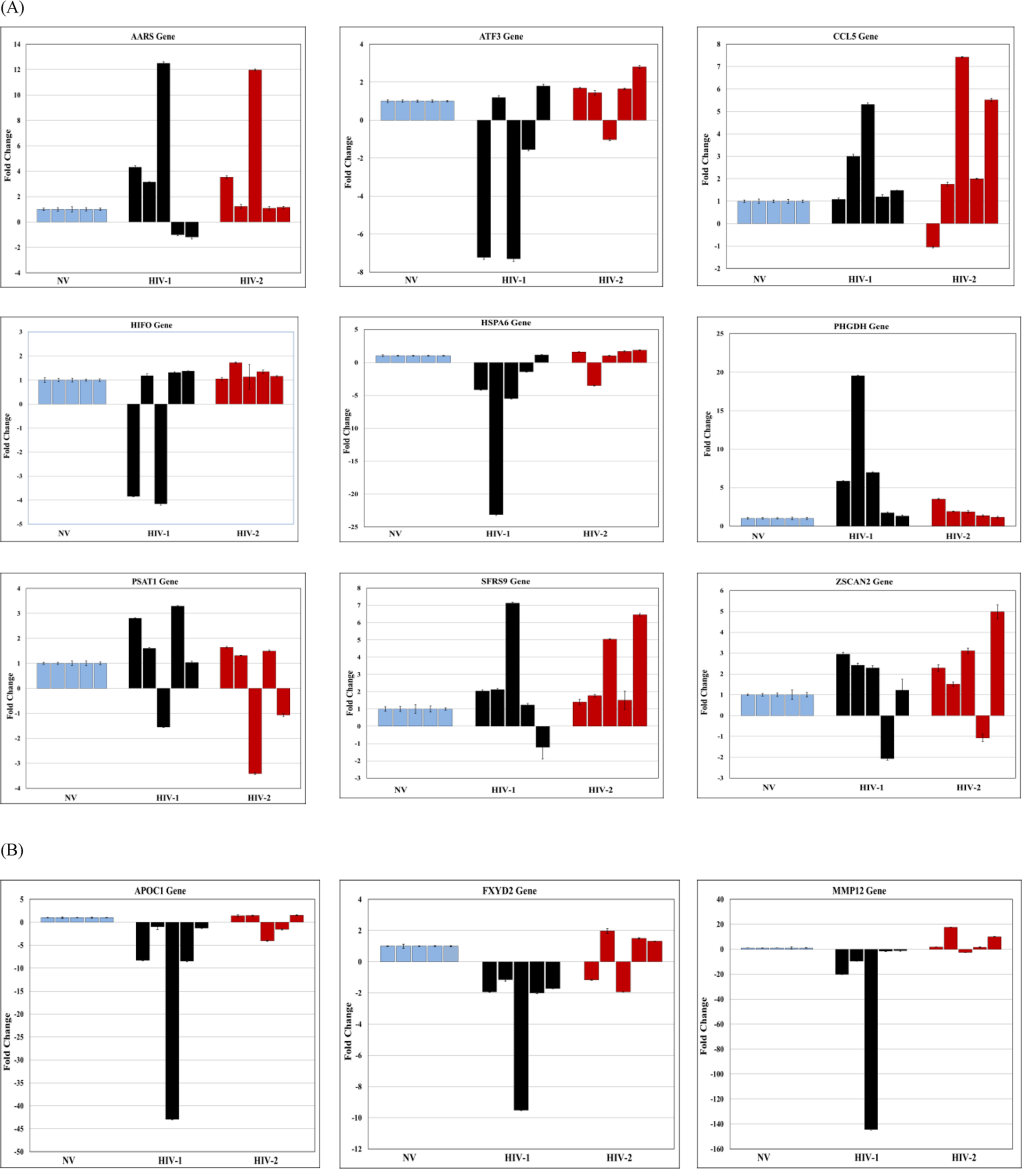
Real-time PCR detection of host genes in PBMCs infected with HIV-1 (MN) or HIV-2 (ROD) fifteen days post infection. The RT-PCR results from RNA isolated from PBMCs infected with HIV-1(MN) and HIV-2 (ROD) *(*A) genes differentially up regulated in HIV-1 and HIV-2 infected cells and (B) genes differentially down regulated in HIV-1 and HIV-2 infected cells. The RT-PCR data are derived from independent experiments using PBMCs isolated from five different donors. Each experimental sample subjected to real- time PCR amplification was normalized relative to the endogenous GAPDH control and the relative amount of target gene quantitated. Final results are expressed as n-fold difference in expression of gene-of-interest relative to GAPDH gene for infected and uninfected cells as n-fold = 2^ − (ΔCt infected −ΔCt uninfected). Each sample was run in triplicate to ensure accurate fold change estimation and the results expressed as mean ±SEM.

**Fig 10:**
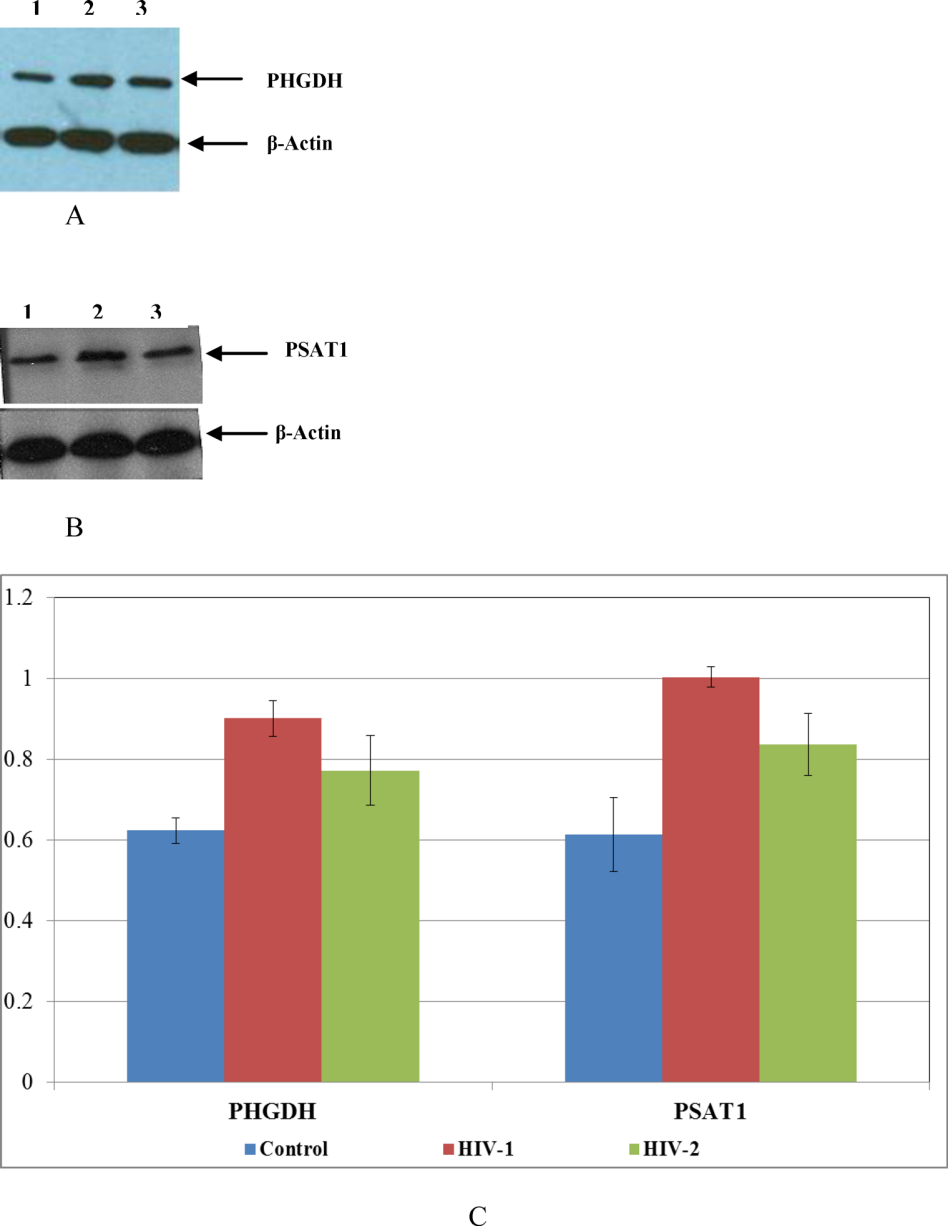
PHGDH and PSAT1 protein expression in PBMC cells infected with HIV-1 and HIV-2 determined by Western blot. (A) PHGDH and (B) PSAT1 protein expression in PBMC cells infected with HIV-1 and HIV-2 determined by Western blot. Lane 1 is denoted as no virus infection control, Lane 2 is denoted as HIV-1 (MN) infected PBMC and Lane 3 is denoted as HIV-2 (ROD) infected PBMC respectively. The data is a representative of three independent experiments. (C) Quantitation of PHGDH and PSAT1 protein expression from Western blot data. The results are derived from independent experiments using PBMCs isolated from three different donors. Each experimental sample subjected to Western Blot analysis was normalized relative to the endogenous β-Actin control and the relative amount of target gene quantitated. The results are expressed as mean ± SEM.

In order to validate the results of the microarray data, a sub-set of differentially expressed genes that demonstrated ˃ 2 fold up regulation, coupled with a low P value (P value ˂ 0.05) were selected for further analysis (Table 3). RNA isolated from PBMC infected with HIV-1 or HIV-2 from 5 independent donors collected at day 7 post-infection was analyzed by real-time PCR. In a similar manner, RNA isolated from Jurkat cells infected with HIV-1 and HIV-2 from 3 independent experiments was used to validate the microarray results. In addition, to determine whether the differential gene expression observed at day 7 post-infection was consistent over a period of time, we also analyzed RNA from PBMC that were cultured for 15 days post-infection. The analysis of real-time PCR results using RNA isolated from PBMC infected with HIV-1 or HIV-2 on day 7 post-infection (Fig 7) revealed an overall agreement with the microarray results, demonstrating that many of the genes were differentially expressed between HIV-1 and HIV-2 infected cells. Variations in the expression pattern of the differentially expressed genes observed in the real-time PCR analysis among the RNA samples isolated from 5 different donors could be due to the inherent differences in the infectivity observed among different donors. Real-time PCR results from RNA isolated from PBMC and Jurkat cells infected with HIV-1 or HIV-2 on day 7 post-infection (Figs 7 and 8) showed that majority of the donor PBMCs and Jurkat cells tested had a consistent pattern of gene expression that validates the microarray results. In HIV-1 infected cells, AARS, ATF3, CCL5, HIF0, PHGDH and PSAT1 genes showed consistent up regulation (100% agreement with microarray data) and APOC1, FADS2, FXYD2, MMP12, KIAA0125 and IL-5 showed consistent down regulation (80–100% agreement with microarray data) similar to the microarray results (Table 3). Other genes like, CBS and CRNDE did not show a consistent pattern of gene expression among the HIV-1infected PBMC donors tested. In HIV-2 infected cells, the genes HSPA6, SFRS9 and ZSCAN2 were found to be differentially up regulated when compared to HIV-1 infected cells validating the microarray results. The differential gene expression observed at day 15 post-infection demonstrated a consistent pattern of gene up regulation of PHGDH and PSAT1 and down regulation of APOC1, FADS2, FXYD2, KIAA0125, and MMP12 over a period of time in HIV-1 infected PBMC (Fig 9A). In HIV-2 infected PBMC only HSPA6 gene was differentially up regulated on day 15 post-infection (Fig 9B) compared to HIV-1 infected PBMC that was consistent with the microarray and day 7 real-time PCR results. Other genes like AARS, ATF3, HIF0, CCL5, FADS2, and FXYD2 were up regulated in HIV-2 infected cells. The expression of PHGDH and PSAT1 genes that showed consistent up regulation were evaluated using Western blot. Western blot data demonstrated a 1.45 and 1.5 fold increased expression of PHGDH and PSAT1 proteins respectively in HIV-1 infected cells (Fig 10), further validating the microarray and real-time PCR results. These genes warrant additional investigations and may be likely candidate biomarkers of infection.

**Table 3:** 

**Day 7—Differentially regulated genes in HIV-1 infected cells**				
**Gene Symbol**	**P-value**	**Adjusted p-value**	**FC Absolute**	**Regulation**
CBS	0.00244966	0.002449663	12.334886	up
SLC7A11	0.022645112	0.022644973	9.18609	up
ULBP1	0.016470283	0.016472566	8.766445	up
H1F0	0.000723184	0.000723186	7.938948	up
PSAT1	0.016851544	0.016851564	6.7728977	up
CRNDE	0.026046049	0.026032747	5.044751	up
PHGDH	0.040211644	0.04021162	5.047805	up
AARS	0.022280922	0.022280919	3.552448	up
ATF3	0.044249944	0.04424989	3.4399707	up
CCL5	0.011322869	0.011322933	2.0074718	up
GRB10	0.030573033	0.030491246	5.1734447	up
FADS2	0.010818841	0.01081885	4.28957	down
MMP12	0.02393175	0.023923703	4.1874332	down
APOC1	0.018256607	0.018256541	3.8645072	down
FXYD2	0.047876157	0.047876118	3.1800854	down
KIAA0125	0.013483486	0.013483494	3.5262792	down
LAIR2	0.020038126	0.020038112	3.259397	down
IL5	0.026236638	0.02624519	2.8544762	down
IL4	0.040611885	0.040611886	2.4296153	down
HIVEP3	0.042159025	0.042159048	2.0655322	down
IL13	0.026788086	0.026788038	1.8714639	down
**Day 7—Differentially regulated genes in HIV-2 infected cells**				
**Gene Symbol**	**P-value**	**Adjusted p-value**	**FC Absolute**	**regulation**
SFRS9	0.01296973	0.012969737	2.221804	up
HSPA6	0.023789361	0.023789438	2.0210264	up
ZSCAN2	0.045484427	0.0454844	1.5853277	up
KRT1	0.020922653	0.020934572	2.161763	down

### Protein-protein network of differentially expressed genes

**Fig 11:**
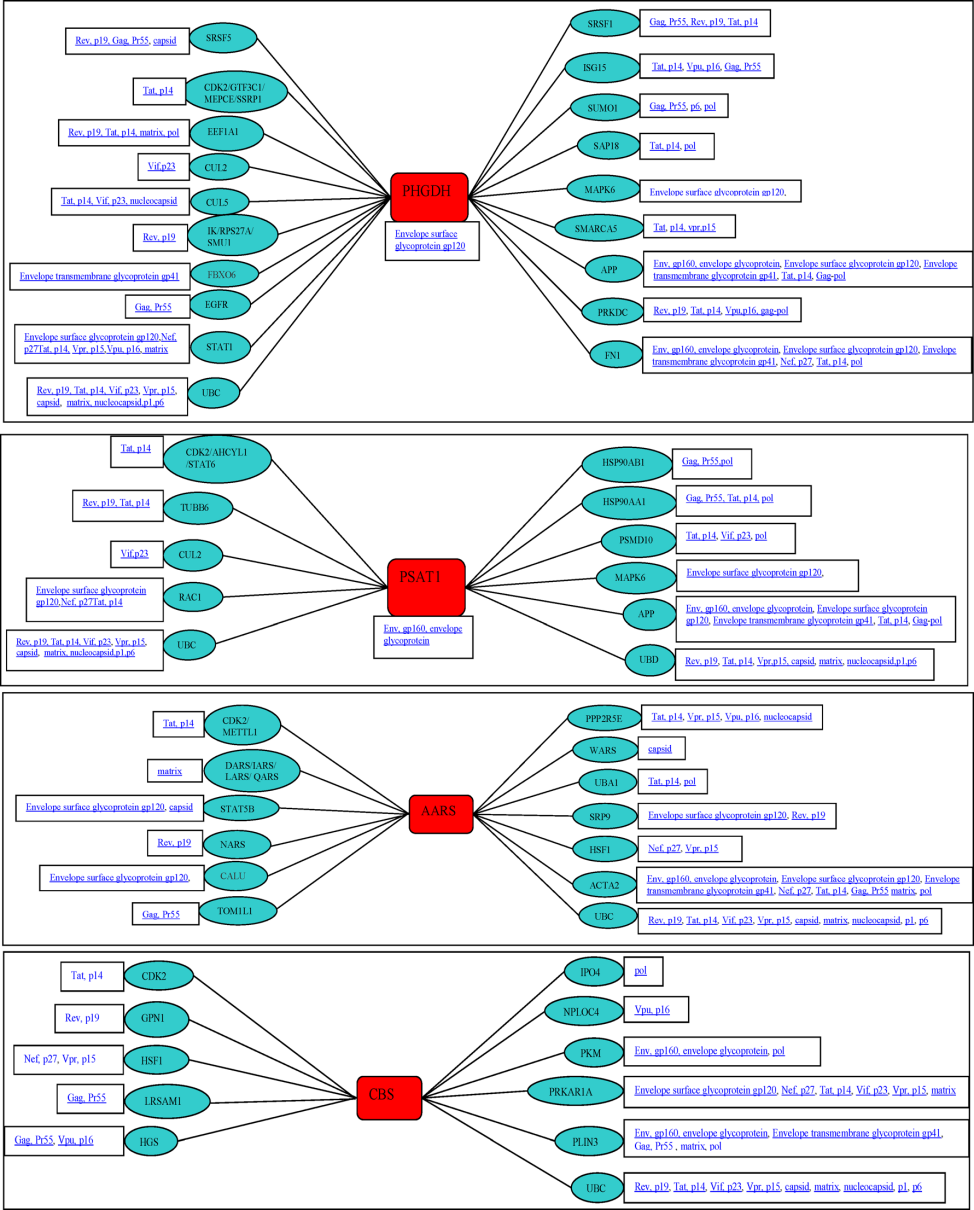
The figure shows interaction of a subset of differentially expressed genes with HIV-1 proteins. The figures were generated using the interaction database form National Center for Biotechnology Information (NCBI) for all genes of interest (GOI) as well as their primary and secondary interaction with HIV-1 proteins.

**Fig 12:**
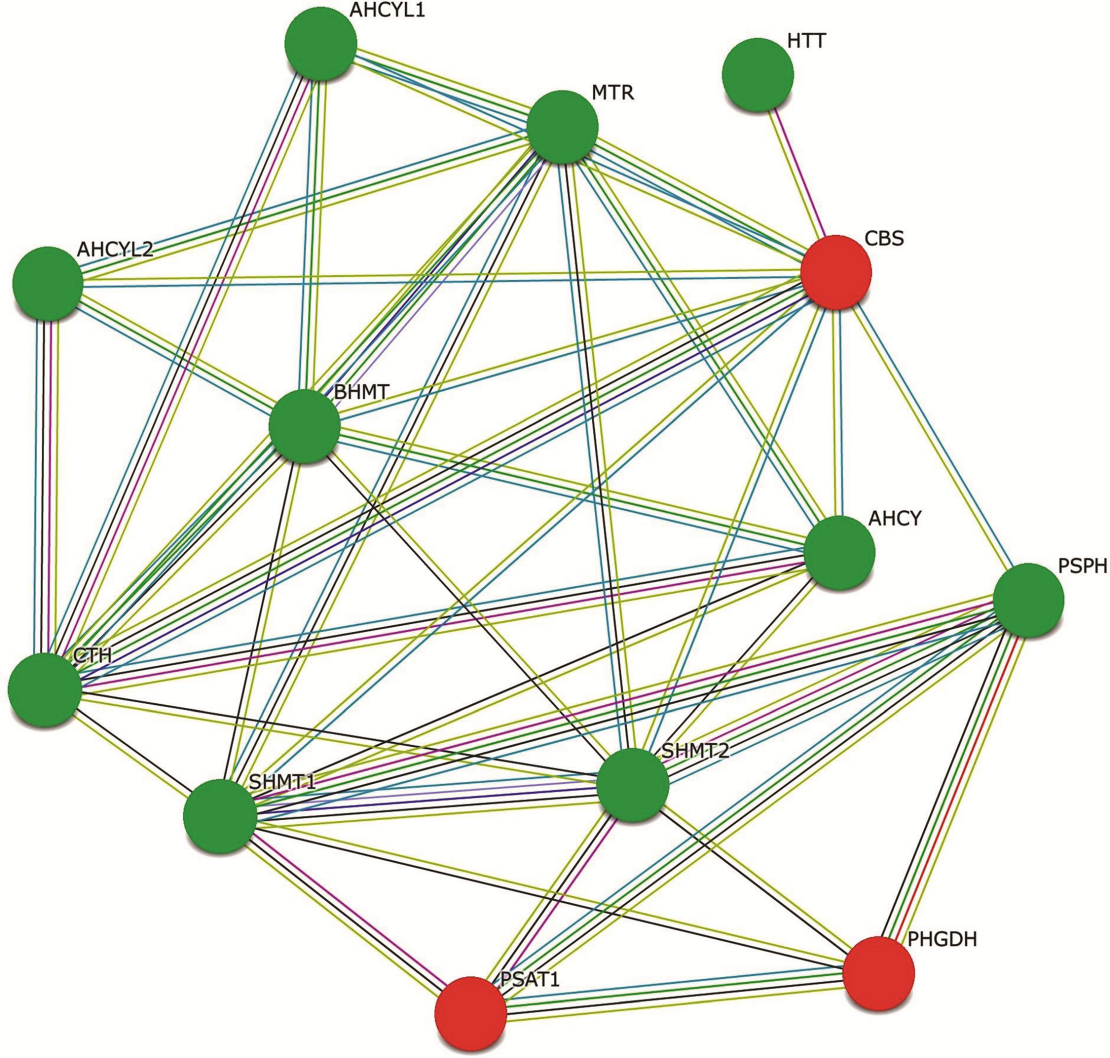
CBS, PHGDH and PSAT1 interactive gene network. The differentially expressed genes CBS, PHGDH and PSAT1 interactive network generated automatically using the STRING database (STRING software 9.1) is shown here. The input differentially expressed genes CBS, PHGDH and PSAT1 are depicted as Red circles. The Predicted Functional Partner proteins depicted as Green circles are given below: PSPH---phosphoserine phosphatase; CTH---cystathionase; MTR 5---methyltetrahydrofolate-homocysteine methyltransferase; BHMT---betaine-homocysteine methyltransferase; SHMT1--- serine hydroxymethyltransferase; AHCY---adenosylhomocysteinase; SHMT2---serine hydroxymethyltransferase 2; HTT---huntingtin; AHCYL2---adenosylhomocysteinase-like 2; AHCYL1---adenosylhomocysteinase-like 1. Multiple Edges color represents: Green---Neighborhood; Red---Gene Fusion; Blue---Co-occurrence; Black---Coexpression; Purple---Experiments; Light Blue---Database; Light Green---Textmining.

**Fig 13:**
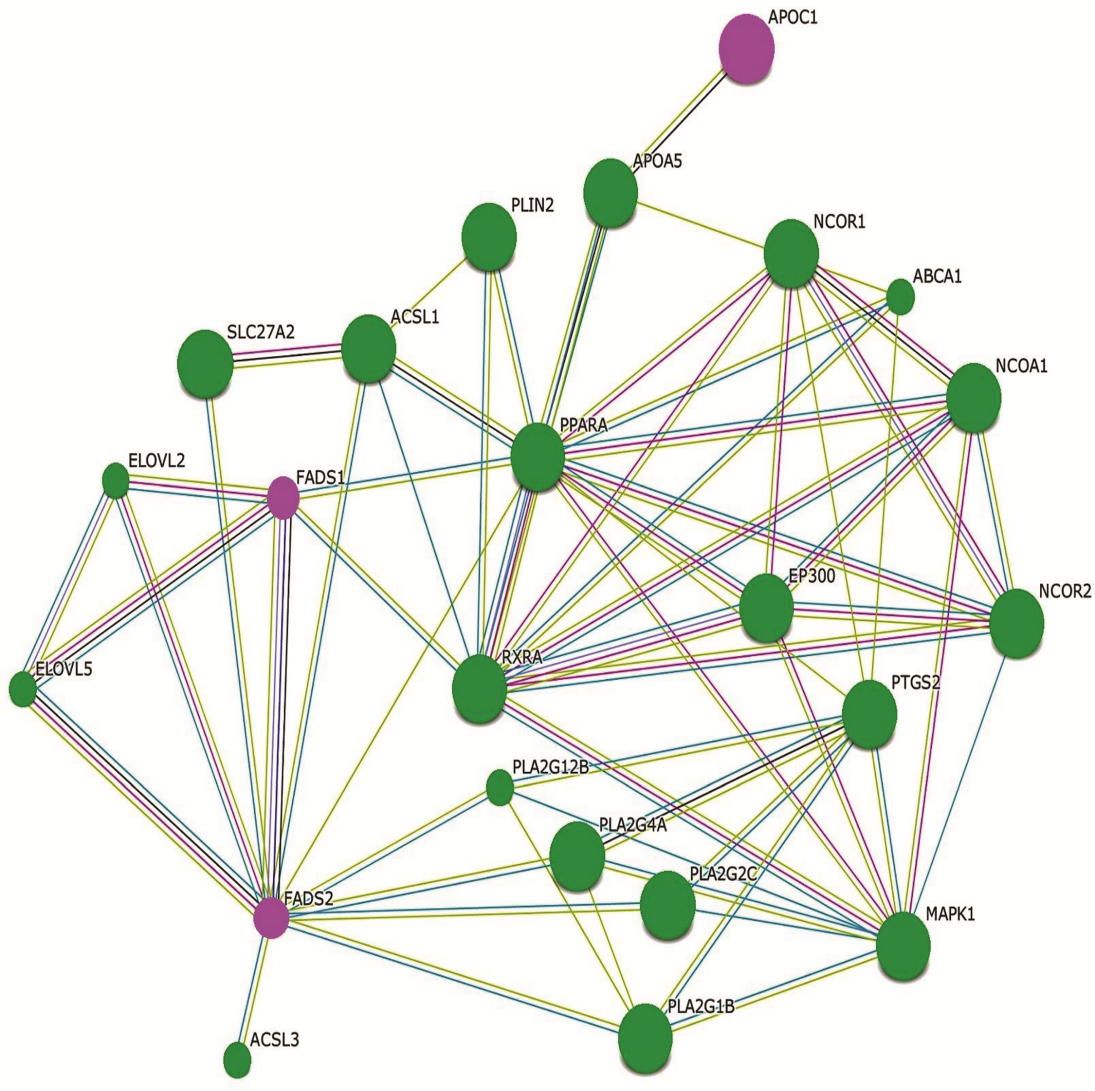
ApoC1, FADS1 and FADS2 interactive gene network. The differentially expressed genes ApoC1, FADS1 and FADS2 interactive network generated automatically using the STRING database (STRING software 9.1) is shown here. The input differentially expressed genes ApoC1, FADS1 and FADS2 are depicted as Purple circles. The Predicted Functional Partner proteins depicted as Green circles are given below: ELOVL2---elongation of very long fatty acids-like 2; ELOVL5---elongation of very long fatty acids-like 5; ACSL1---acyl-CoA synthetase long-chain family member 1; PPARA---peroxisome proliferator-activated receptor alpha; PLA2G1B ---phospholipase A2, group IB (pancreas); PLA2G12B ---phospholipase A2, group XIIB; SCL27A2---solute carrier family 27 (fatty acid transporter); ACSL3---acyl-CoA synthetase long-chain family member 3; PLA2G4A ---phospholipase A2, group IVA (cytosolic, calcium dependent); PLA2G2C ---phospholipase A2, group IIC. Multiple Edges color represents: Green---Neighborhood; Red---Gene Fusion; Blue---Co-occurrence; Black---Coexpression; Purple---Experiments; Light Blue---Database; Light Green---Textmining.

**Fig 14:**
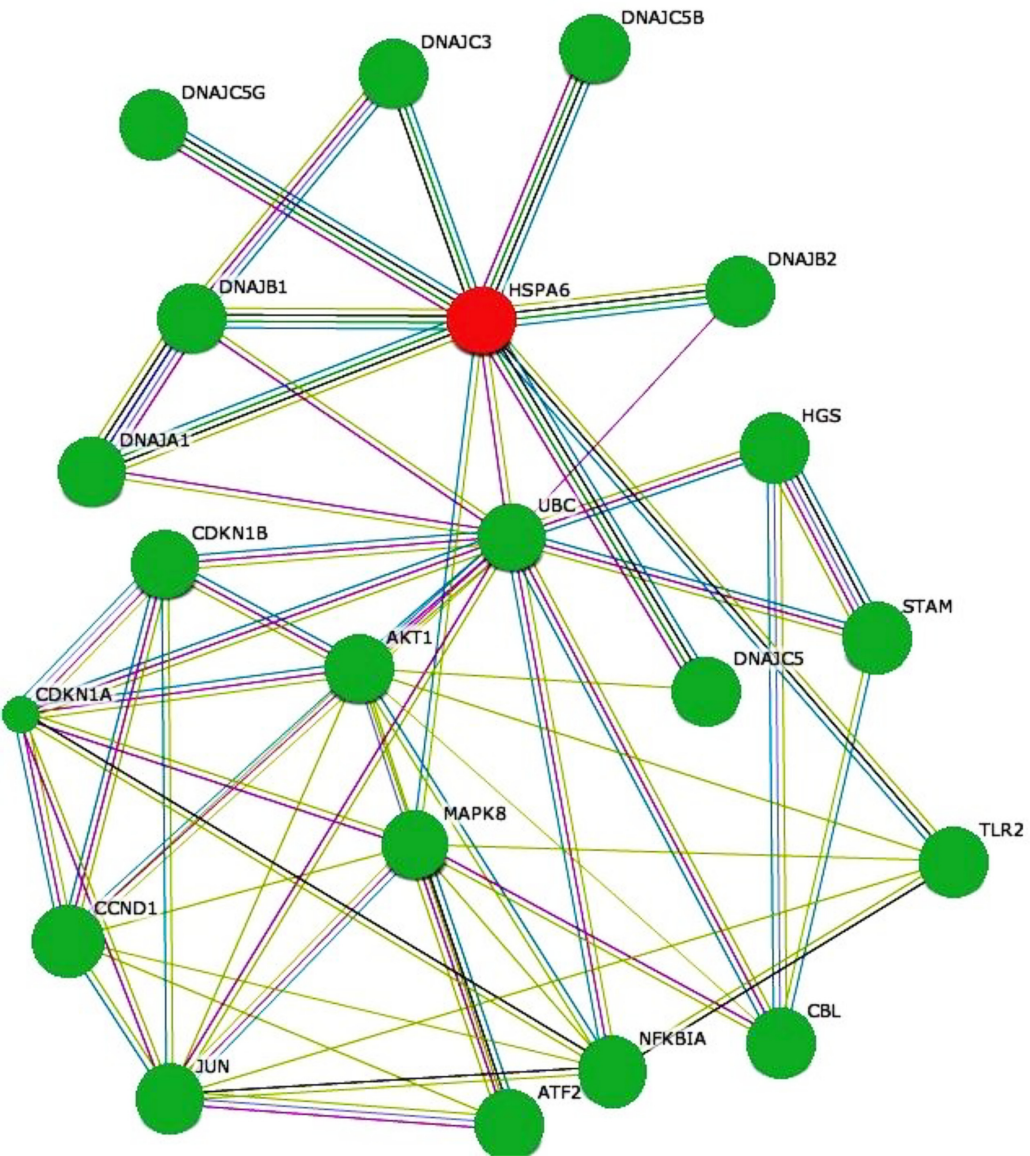
HSPA6 interactive gene network. The interactive network generated automatically using the STRING database (STRING software 9.1) for heat shock protein 6 (HSPA6) gene that was differentially upregulated in HIV-2 infected cells is shown here. The input differentially expressed gene HSPA6 is depicted as Red circles. Predicted Functional Partners proteins depicted as Green circles are given below: DNAJB1---DnaJ (Hsp40) homolog, subfamily member 1; UBC---Ubiquitin C; MAPK8---mitogen-activated protein kinase 8; DNAJA1---DnaJ (Hsp40) homolog, subfamily member A; DNAJB2---DnaJ (Hsp40) homolog, subfamily member B; TLR2---Toll-like receptor 2; DNAJC5---DnaJ (Hsp40) homolog, subfamily member C; DNAJC5G ---DnaJ (Hsp40) homolog, subfamily member C; DNAJC5B ---DnaJ (Hsp40) homolog, subfamily member C; DNAJC3---DnaJ (Hsp40) homolog, subfamily member C. Multiple Edges color represents: Green---Neighborhood; Red---Gene Fusion; Blue---Co-occurrence; Black---Coexpression; Purple---Experiments; Light Blue---Database; Light Green---Textmining.

Fig 11 shows interaction of a subset of differentially expressed genes AARS, CBS, PHGDH and PSAT1 with HIV-1 proteins. The figures were generated using the interaction database form National Center for Biotechnology Information (NCBI) for all genes of interest (GOI) as well as their primary and secondary interaction with HIV-1 proteins. Fig 11 provides additional insight into the probable mode of action of the differentially expressed genes in modulating HIV infection. To better understand the relationships of the differentially expressed genes in serine family amino acid biosynthesis process and lipid biosynthesis process pathways, we examined protein-protein interactions between protein products of all differentially expressed genes in these two pathways based on the STRING database. The protein-protein network is shown in Figs 12, 13 and 14. Our results indicate that in HIV-1 infected cells, genes coding for enzymes involved in aspects of the amino acid biosynthesis (CBS, PHGDH, PSAT1 and PSPH) that were differentially expressed form a close network of interacting proteins (Fig 12). Similarly enzymes involved in fatty acid biosynthesis process (FADS1, FADS2 and APOC1) also form a network of interconnected proteins (Fig 13). The results from the STRING database for heat shock protein 6 (HSPA6) gene that was differentially upregulated in HIV-2 infected cells (Fig 14), indicates that HSPA6 gene which facilitates proper folding and stabilization of newly synthesized polypeptides in cytosol and organelles, interacts with other proteins that function as chaperones that help in protein folding and membrane trafficking. HSPA6 also interacts with ubiquitin C (UBC), an enzyme in the ubiquitin pathway. This could explain both how the deregulation of key ‘‘hub” genes may affect multiple pathways, and also how deregulation of distinct host genes may modulate HIV infection.

## Discussion

HIV-1 and HIV-2 are closely related retroviruses that share considerable similarities in their genome architecture, infectivity and modes of transmission, cytopathic traits, viral replication and pathogenesis. Clinically, both HIV-1 and HIV-2 infection cause AIDS but the major difference between the two infections is the slower disease progression observed in HIV-2 infection [2, 3, 9]. The goal of our study was to find insights into the major differences in pathogenesis and disease progression observed between HIV-1 and HIV-2. Previously, we examined differences in apoptotic signaling pathways during infection with either HIV-1 or HIV-2 in Jurkat cells. HIV-1 infection generated more reactive oxygen species (ROS) and increased the expression of diverse molecules involved in cell signaling [11, 13]. In addition, previous studies demonstrated that HIV-1 infection induced greater cell death, activation of apoptosis and autophagy relative to HIV-2 infection. Overall, infection with HIV-1 or even treatment with HIV-1 gp120 alone could induce major apoptotic pathways, suggesting that HIV-1 infection regulates the apoptosis process to facilitate viral replication and promote pathogenesis [10, 12].

To further examine the observed differential host responses to infection with HIV-1 or HIV-2, we wanted to use other molecular and cellular approaches like proteomics, microarray and PCR-array based techniques that facilitate a global comparison of gene expression patterns between different groups of samples. Therefore, in an attempt to characterize the role played by host factors leading to the observed differences in pathogenesis and disease progression between HIV-1 and HIV-2, we analyzed the effects of HIV-1 (MN) and HIV-2 (ROD) infection on the expression of host factors in PBMC at the RNA level using the Agilent Whole Human Genome Oligo Microarray. The gene expression profiling analysis identified a subset of differentially expressed genes in HIV-1 and HIV-2 infected cells (Table 3). Host gene expression profiles were significantly changed at the time point tested (day 7). Functional analysis of the differentially expressed genes in HIV-1 and HIV-2 infected cells using the Gene Ontology database (GO) revealed significant differences in the biological and molecular functions of the genes. Significant differences that were identified in HIV-1 infected cells were related to genes whose characteristic functions involved regulation of transcription, immune cell activation and proliferation, cell cycle, apoptosis and cellular metabolism. While up regulation of apoptosis related genes alone does not lead to induction of apoptosis in the absence of stimuli, our current findings taken together with previous studies demonstrating HIV-1 infection induces changes in proteins related to major apoptotic pathways, indicates that the observed up regulation of positive regulators of apoptosis in response to HIV-1 infection contributes to the relative increase in apoptosis and cell death seen in HIV-1infected cells relative to HIV-2 infection [11, 12]. HIV infection is known to cause dysregulation of the cell cycle by hijacking the cellular transcription machinery to promote viral replication and proliferation. Consistent with reports in literature, we observed a number of the differentially expressed genes in HIV-1 infected cells that were involved in the modulation of transcription, cell division, proliferation and cell activation compared with HIV-2 infected cells. Genes like ATF3, BHLHE41, CREBBP, CBX4, EGR2, and TGIF1 that were found to be up-regulated by the microarray analysis are mainly involved in regulation of transcription. The ATF3 protein that belongs to the cAMP responsive element-binding (CREB) family of transcription factors and the CREB binding protein (CREBBP) that functions as a co-activator of transcription are known to influence binding to the CRE sequence present in many viral and cellular promoters thereby modulating host gene expression, HIV-1 transcription and viral replication. Current literature indicates that induction of ATF3 is believed to affect cell death and cell cycle progression. Our results indicate that ATF3 and other transcription regulators like CBX4, EGR2, and TGIF1 and Basic helix-loop-helix family member E41 (BHLHE41) were up-regulated only in HIV-1 infected cells. These factors are known to exert a modulatory effect on gene expression and play a critical role in cell cycle progression. Whereas in HIV-2 infected cells no such genes were found to be up regulated, the only genes found to be up regulated were related to viral reproduction, nuclear transport and virus-host interactions (CTBP2, THOC4, NUPL2 and ZSCAN2). Taken together, these results imply that differential expression of these genes observed in HIV-1 infection may lead to the significant differences observed in HIV-1 infection. The establishment of immune activation and inflammation during the course of HIV infection involve several mechanisms related to viral replication, proliferation, cell death, and secretion of cytokines and chemokines. Most HIV-2 infected individuals have low viral loads and a slower rate of disease progression compared with HIV-1 infected individuals. One explanation may be that HIV-2 infected individuals are capable of mounting robust immune responses (26) and display significantly less immune activation (27) when compared with HIV-1 infected individuals. Our results indicate that genes involved in immune cell proliferation, immune response, and negative regulation of pro-inflammatory cytokines (APOE, HLA-DMB, HLA-DOB, IL-4, IL-5, IL-13 and CTNNB1) were all down-regulated only in HIV-1 infected cells, whereas genes related to these functions were not down-regulated in HIV-2 infected cells. The differential regulation of genes involved in immune response and inflammation between HIV-1 and HIV-2 infections may account for the more rapid disease progression seen in HIV-1 infected patients.

Immune activation and inflammation associated with chronic HIV infection can modulate cellular metabolism [29, 30]. During the course of HIV infection, the growth, proliferation, differentiation and functions of the immune cells to promote antiviral responses cause an increased metabolic demand on the cell leading to metabolic disturbances [31]. Our study indicates that major cellular pathways involved in the biosynthesis of lipids and steroids and amino acids were impacted by infection with HIV-1. In our study genes involved in lipid and steroid metabolism (FADS1, FADS2, FABP5, FDXR, EBP, CYP51A1, DHCR7, SCARB1, AGPAT4, APOE, and APOC1) were found to be significantly down regulated in HIV-1 infected cells only. Comparison of our data set from HIV-1 infected PBMC to the differentially expressed genes in GSE2171 data set [18] and to the differentially expressed genes in GSE6740 data set [28] has identified that fatty acid binding protein 5 (FABP5) was down modulated in all 3 studies. KEGG analysis and String Interaction Network analysis indicate the close association of enzymes FADS1, FADS2 and APOC1 involved in lipid and steroid metabolism (Table 2 and Fig 13). Lipid and cholesterol abnormalities are a common finding in HIV infected persons [32–34]. Similarly, genes involved in cellular biosynthesis and amino acid metabolism (AARS, CBS, PHGDH and PSAT1) were up regulated in HIV-1 infected cells. Whereas, genes related to these functions were not differentially regulated in HIV-2 infected cells. In addition, the differently expressed genes and their co-expression network clusters in HIV-1 infected PBMC demonstrated significant enrichment for the GO biological process terms such as lymphocyte mediated immunity, steroid metabolic process, organelle assembly, regulation of protein serine / threonine kinase activity and serine family amino acid metabolic process that are distinct from the GO biological process terms identified in HIV-2 infected PBMC.

Of particular interest were genes PHGDH and PSAT1, found to be consistently up regulated in HIV-1 infected cells. KEGG analysis and String Interaction Network analysis indicate the close association of the two enzymes PHGDH and PSAT1 (Table 2 and Fig 12). These proteins are enzymes involved in the serine biosynthesis pathway which converts the glycolytic intermediate 3-phosphoglycerate into serine. Serine is a central metabolite involved in the biosynthesis of other amino acids, nucleotides and alpha-ketoglutarate (α-KG) another key metabolic intermediate from the break-down of glutamate catalyzed by PSAT1. The enzyme, phosphoglycerate dehydrogenase (PHGDH) is the first enzyme branching from glycolysis in the serine biosynthetic pathway. Similarly, phosphoserine aminotransferase (PSAT1), is an enzyme that catalyzes the reversible conversion of 3-phosphohydroxypyruvate to phosphoserine and of 3-hydroxy-2-oxo-4-phosphonooxybutanoate to phosphohydroxythreonine. In actively proliferating cells, for the TCA cycle to continue uninterrupted during increased macromolecular biosynthesis, the cell must replenish the depleted pools of metabolic intermediates (anaplerosis). Therefore, intermediates of the TCA cycle like α-KG are diverted to the TCA cycle as biosynthetic precursor to up regulate anaplerotic reactions [35, 36]. The serine biosynthesis pathway, involving PHDGH and PSAT1 is implicated in contributing a significant fraction of glutamate to the α-KG flux, thereby playing an important role in TCA anaplerosis [37]. Recent evidence has shown that PHGDH is amplified and over-expressed in certain types of melanomas and breast cancer and plays a key role in cancer metabolism by influencing proliferation and metastases [37–40]. Reports have also implicated PSAT1 and PHGDH as having direct interaction with HIV-1 gp120 envelope protein [41, 42]. The predicted models of PHGDH and PSAT1 interaction with other cellular gene products and pathways (Fig 12) indicate a central role for these enzymes in helping the cell to adapt to metabolic changes in response to HIV-1 infection. While the exact function of these genes in modulating HIV-1 replication and life-cycle is not known, we believe that in HIV-1 infected cells, high PHGDH and PSAT1 expression might contribute to the serine pathway flux in replenishing metabolic intermediates of the TCA cycle thus promoting cellular survival and proliferation.

The present study has identified several differentially expressed host genes in response to HIV-1 and HIV-2 infections. This is a significant step towards the identification of a set of biomarkers based on host genomic foot prints that may become powerful tools in defining the pathogenesis of diverse HIV variants, especially when coupled with classic markers, such as CD4+ T cell count and viral load. Finally, identification of the differences in specific host metabolic and biosynthetic pathways impacted by HIV-1 or HIV-2 infection may lead to the use of more effective and targeted therapies for each of these HIV types than currently available. Further evaluation of these prognostic bio-markers in HIV-1 and HIV-2 infections is warranted. In the future, we plan to extend these studies to identify bio-markers that may be differentially regulated in HIV-1/HIV-2 infection using patient derived T-lymphocyte and macrophage systems and clinical isolates.

## Supporting Information

S1 File Excel file containing differentially expressed genes. (XLS)

S2 File Excel file containing GO Term enrichment for the differentially expressed genes. (XLS)

S1 Table Comparison of Day 7—differentially regulated genes in HIV-1 infected cells among experimental and two data sets from Gene Expression Omnibus (GDS 2649 and GDS 1449). Gene expression profile in HIV-1 and HIV-2 infected PBMC, Accession # GSE68563 [NCBI tracking system #17333911].
(DOC)
