# Supplementary material for: Analysis of Host Gene Expression Profile in HIV-1 and HIV-2 Infected T-Cells
Source: PLoS One. 2016 Jan 28;11(1):e0147421. doi: 10.1371/journal.pone.0147421 (PMC4731573; doi:10.1371/journal.pone.0147421)
Supplement: S1 Table — Gene expression profile in HIV-1 and HIV-2 infected PBMC, Accession # GSE68563 [NCBI tracking system #17333911]. (DOC) [file pone.0147421.s003.doc]

**S1 Table**

| **Comparison of Day 7 - differentially regulated genes in HIV-1 infected cells among experimental and two data sets from Gene Expression Omnibus (GDS 2649 and GDS 1449)** | | | |
| --- | --- | --- | --- |
| **Gene Symbol** | **FC** | **FC GDS 2649** | **FC GDS 1449** |
| CBS | 12.334886 | 0.8413 | 1.341796733 |
| SLC7A11 | 9.18609 | 1.212441315 | 1.279042384 |
| ULBP1 | 8.766445 | 1.005933118 | 1.68343416 |
| H1F0 | 7.938948 | 1.086956522 | 0.694031985 |
| PSAT1 | 6.7728977 | 1.177017322 | 1.018915307 |
| CRNDE | 5.044751 | N/A | N/A |
| PHGDH | 5.047805 | 1.138650111 | 1.122759401 |
| AARS | 3.552448 | 1.126803853 | 1.093504001 |
| ATF3 | 3.4399707 | 1.174403263 | 0.411748207 |
| CCL5 | 2.0074718 | 1.192135677 | 2.310732643 |
| GRB10 | 5.1734447 | 1.335846686 | 1.270107676 |
| FADS2 | 0.2331236 | 1.056397306 | 0.254472406 |
| MMP12 | 0.23880978 | 1.124502353 | 1.329048394 |
| APOC1 | 0.258765206 | 0.831122342 | 1.889181979 |
| FXYD2 | 0.314456964 | 1.091186123 | 1.447015834 |
| KIAA0125 | 0.283585032 | 0.937161881 | N/A |
| LAIR2 | 0.306805216 | 1.343687597 | 1.296886488 |
| IL5 | 0.350326971 | 1.523809524 | 1.472161357 |
| IL4 | 0.411587793 | 0.708666667 | 1.371388001 |
| HIVEP3 | 0.484136728 | 2.425026969 | 1.402784213 |
| IL13 | 0.534341058 | 1.349562081 | 1.441101695 |

FC: Fold Change

N/A: Not applicable
